# Around the Hospitals

**Published:** 1893-03-18

**Authors:** 


### AROUND THE HOSPITALS.

**The General Infirmary, Leeds,** at the annual meeting afforded statistics which testified to the wisdom of the extension of the institution. It was supposed at first that it would not be necessary to place all the extra accommodation into requisition. But facts have proved otherwise, and an increase of 347 in-patients during the past year made it a very busy one. The Treasurer, Mr. Jowitt, pointed out that this increase of the Infirmary will require the addition of £3,000 in annual subscriptions. The new buildings are paid for, and it is to be expected that an institution in a district which so imminently needs, will be as well supported as it deserves.

**The Royal Free Hospital,** Gray's Inn Road, has since the annual meeting of 1892 received the Royal Charter of Incorporation. A very earnest desire is now felt to place the hospital on the best basis, and it is hoped that the work of reconstruction will be commenced in May. The sum of £7,634 now stands to the credit of the Building Fund, and the plans of Mr. W. Harvey, architect to the hospital, have been approved. These plans include a laundry, and the estimate amounts to £15,000, which shows that the balance of over £7,000 has yet to be collected. This we hope will speedily be forthcoming. The institution does good work and has many friends, and the record of the past year has been most successful, the receipts nearly doubling those of the past year. Sir Charles Hall has consented to become a member of the Committee, and Lord Saye and Seal to be Vice-President.

**The New Hospital for Women,** Euston Road, was congratulated by Mrs. Garrett Anderson, at the annual meeting, on now being able to walk alone. The patients' subscriptions have increased greatly, and the institution generally shows signs of great activity and success. Upwards of 500 in-patients have been admitted during the year, whilst in the out-patients department 21,866 attendances are recorded. Sixteen patients were received into the separate pay wards, and the Samaritan Fund has greatly assisted patients by enabling them to enter convalescent homes.

**The Newcastle Infirmary** is about to issue a special appeal for funds, and after the annual meeting of the institution, a meeting of working men and governors was held to decide upon the best means of raising the required sums. It has been proposed that a house to house collection should be made, and that the Hospital Sunday and Saturday funds and other medical charity funds should be induced to increase their subscriptions to the hospital. The number of patients during the past year was practically the same as that of the previous year, whilst a slight reduction per head was made in the cost of the patients.

**King's College Hospital** has been better supported during the past year than previously, but yet grave cause for anxiety remains as to meet the expenditure, still largely in excess of the income. The remaining stock belonging to the institution had to be sold. The hospital has, therefore, no reserve fund to fall back upon during the present year, and is therefore urgently in need of special and substantial help. The number of patients both in and out show a considerable increase, and there is no doubt that the hospital accomplishes a large amount of useful work, whilst the management is both careful and economical, the accounts showing a decrease of £230 in the expenditure during the past year, in spite of the larger number of patients treated.

